# Synthesis, Characterization, and Bacterial Fouling-Resistance Properties of Polyethylene Glycol-Grafted Polyurethane Elastomers

**DOI:** 10.3390/ijms20041001

**Published:** 2019-02-25

**Authors:** Iolanda Francolini, Ilaria Silvestro, Valerio Di Lisio, Andrea Martinelli, Antonella Piozzi

**Affiliations:** Department of Chemistry, Sapienza University of Rome, 00185 Rome, Italy; ilaria.silvestro@uniroma1.it (I.S.); valerio.dilisio@uniroma1.it (V.D.L.); andrea.martinelli@uniroma1.it (A.M.)

**Keywords:** segmented polyurethanes, polyethylene glycol, microbial biofilm, antifouling materials, medical device-related infections, wound dressings

## Abstract

Despite advances in material sciences and clinical procedures for surgical hygiene, medical device implantation still exposes patients to the risk of developing local or systemic infections. The development of efficacious antimicrobial/antifouling materials may help with addressing such an issue. In this framework, polyethylene glycol (PEG)-grafted segmented polyurethanes were synthesized, physico-chemically characterized, and evaluated with respect to their bacterial fouling-resistance properties. PEG grafting significantly altered the polymer bulk and surface properties. Specifically, the PEG-grafted polyurethanes possessed a more pronounced *hard/soft* phase segregated microstructure, which contributed to improving the mechanical resistance of the polymers. The better flexibility of the *soft* phase in the PEG-functionalized polyurethanes compared to the pristine polyurethane (PU) was presumably also responsible for the higher ability of the polymer to uptake water. Additionally, dynamic contact angle measurements evidenced phenomena of surface reorganization of the PEG-functionalized polyurethanes, presumably involving the exposition of the polar PEG chains towards water. As a consequence, *Staphylococcus epidermidis* initial adhesion onto the surface of the PEG-functionalized PU was essentially inhibited. That was not true for the pristine PU. Biofilm formation was also strongly reduced.

## 1. Introduction

Polymeric materials have a prominent place in biomedical applications, due to their broad range of physico-chemical properties that can be tailored to fit a wide plethora of applications [1,2,3]. Segmented polyurethanes (PUs) are among the most important classes of biomedical polymers, mainly due to their excellent hemocompatibility and unique mechanical properties deriving from the presence of *hard* segment-rich and *soft* segment-rich domains in a phase-separated microstructure. Such *hard/soft*-phase segregation in the polymer permits the combination of elastomeric properties that are typical of rubbers, with high mechanical resistance properties typical of thermoplastic materials [4]. A large variety of biomedical-grade PUs with different compositions, such as Biomer®, Pellethane®, and Cardiothane, are on the market, and their applications cover mainly cardiovascular devices, including central venous catheters and heart valves, but also artificial organs, scaffolds for tissue engineering, and wound dressings [5].

Despite the benefits of using PUs for intravascular devices manufacturing, complications are still associated with their use. Especially, these materials do not protect patients from the risk of developing localized or systemic bloodstream infections [6,7]. For this reason, considerable efforts have been made over the years to improve the bacterial fouling resistance of polyurethanes, in order to reduce the incidence of bloodstream infections [8]. Traditionally, antimicrobial or antifouling polyurethanes were obtained by the adsorption/conjugation of drugs or antiseptics [9,10,11,12,13]. More recently, research efforts have been focused on either the use of natural compounds with anti-biofilm properties [14,15,16,17], or on the development of intrinsically antimicrobial and antifouling materials, by either physical or chemical technological approaches [17,18,19,20]. Physical approaches mainly consist of developing micro- or nano-scale surface texturing in order to affect bacterial adhesiveness, growth, and more in general, biofilm formation [21,22,23,24]. Chemical approaches, instead, mainly involve the functionalization of material surfaces, to meet some criteria that are well-recognized to confer repelling activities, which include strong hydrophilicity, neutral charge, and the presence of groups that are able to establish hydrogen bonds [25].

Polyethylene glycol (PEG) is undoubtedly, the most closely investigated antifouling polymer, as it meets all of the criteria listed above [26]. The ability of such a polymer to resist the adsorption of proteins and bacteria has been related to both hydration and steric hindrance effects [27]. Specifically, in aqueous environment, the hydration of PEG chains occurs resulting in the formation of a layer consisting of tightly bound water molecules, which acts as a physical barrier (steric repulsion) against the approach of proteins and bacteria to the polymer itself [28]. Measurements of forces of interaction between bacteria and PEG chains anchored onto glass surfaces showed that PEG brushes not only blocked the long-range attractive forces, but also introduced repulsive steric effects between the bacteria and the substrate. Presumably, the repulsive forces resulted from the compression of the highly flexible PEG chains, which would involve the removal of water molecules from the hydrated polymer, and is not a thermodynamically favorable process [29].

As for polyurethanes, PEG was either introduced in the backbone as a *soft* segment [30,31,32], or grafted in the polymer side chain [33,34]. Most of such PEG-containing PUs were studied in terms of bio- and hemo-compatibility properties, through the study of polymer affinity towards biomolecules such as albumin, fibrinogen, or heparin [35,36,37,38,39]. Only recently, a series of studies has been carried out to investigate the influence of PEG-containing PUs on microbial adhesiveness [22,40,41]. The few positive datasets available so far encourage further experimentations in order to uniquely confirm PEG as a potent antibacterial fouling agent for polyurethane surfaces.

In this study, a segmented carboxylated polyurethane, obtained by the polymerization of an aromatic di-isocianate, an ether macrodiol, and a low molecular weight diol displaying a carboxylic group, was functionalized with PEG by a Steglich esterification reaction, in order to improve polymer-antifouling abilities. Specifically, PEG was grafted onto the polymer aromatic *hard* segments, which, being hydrophobic, were supposed to be the domains that were more susceptible to bacterial colonization. We hypothesized that the reduction in the *hard* domains’ hydrophobicity by PEG grafting could be a potential strategy to confer antifouling features to the polymer without changing the polymer backbone composition, neither in terms of monomer type (aliphatic vs. aromatic) nor in *hard/soft* segment ratio, which could have had a negative effect on the polymer physico-mechanical properties, as previously reported [42]. The obtained PEG-grafted PUs were fully characterized, to assess the effect of PEG-grafting on the thermal and mechanical properties of the polymer itself. Additionally, contact angle measurements and experiments with water uptake were carried out to verify the influence of the polymer bulk and surface hydrophilicity on the adhesiveness and biofilm formation of *Staphylococcus epidermidis*, a bacterial strain chosen because of its implication in the pathogenesis of intravascular device-related infections.

## 2. Results and Discussion

**Figure 1:**
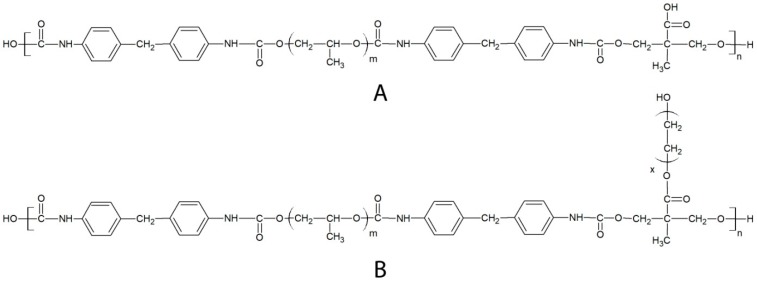
Chemical structure of the repeat unit of PEUA (**A**) and PEUA-PEG (**B**).

In this study, a segmented polyurethane (PEUA, Figure 1)-containing polypropylene oxide (PPO) as a *soft* phase, and the aromatic methylene bis-phenyl-diisocyanate (MDI), plus di-hydroxymethyl propionic acid (DHMPA) as a *hard* phase, were synthesized and functionalized with poly(ethylene glycol), with the aim of obtaining polymers (PEUA-PEG, Figure 1) that were resistant to bacterial adhesion. PEUA is a hemo-compatible polymer, which was synthesized for the first time by our group [43], and it contains interesting elastomeric properties and biocompatibilities. This polymer was also shown to be able to bind antibiotics [44] and complex antiseptic metal ions [45], thanks to the presence of a reactive carboxylic group per repeat unit. Such a group was now exploited for the grafting of PEG by a Steglich reaction mediated by di-cyclohexylcarbodiimide and 4-dimethylaminopyridine (DMAP) activation. 

Specifically, three PEG:PEUA molar ratios (2:1, 3:1, and 5:1) were employed during synthesis, and an excess of PEG was chosen to reduce the probability of PEUA cross-linking by the bi-functional PEG. Among the three obtained PEG-functionalized polymers, the one resulting from a 2:1 PEG:PEUA molar ratio was found to be insoluble in organic solvents, and it also precipitated during functionalization, suggesting a high degree of polymer cross-linking. In contrast, a good degree of solubility in all common organic solvents (tetrahydrofurane, dimethyl formamide, and dimethyl sulfoxide) was shown by the polymer obtained from a 5:1 molar ratio while the polymer obtained with a 3:1 molar ratio showed intermediate behavior, resulting in only partial solubility in some solvents, specifically in di-methylformamide. 

**Figure 2:**
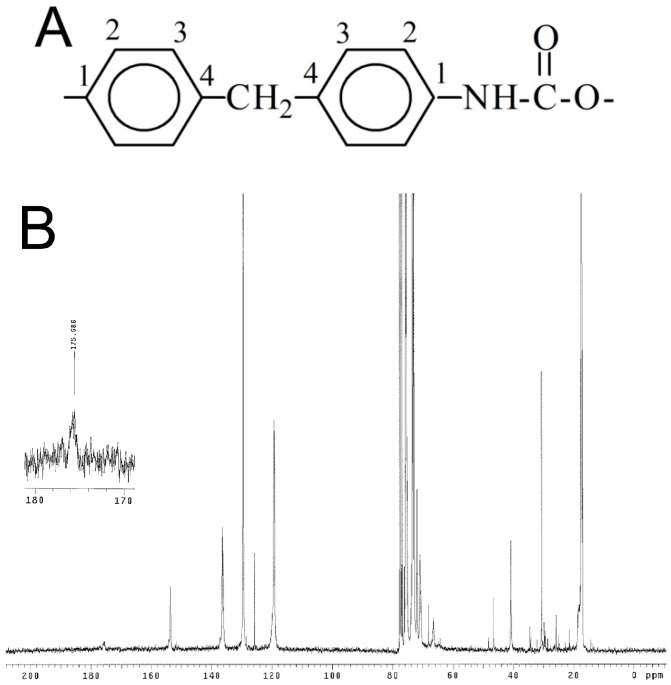

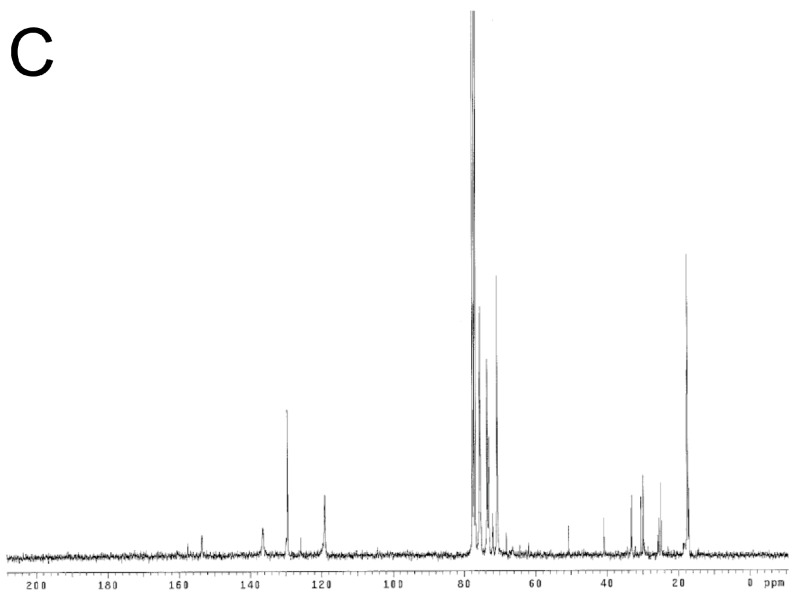
Numeration of methylene bis-phenyl-diisocyanate (MDI) carbons in PEUA (**A**); ^13^C-NMR spectrum of PEUA (**B**) and PEUA-PEG obtained with a 5:1 PEG:PEUA molar ratio (**C**). In the inset of B, the magnification of the signal at 175 ppm, attributed to the PEUA carboxylic group, is reported.

Nuclear magnetic resonance spectroscopy was used to evaluate the success of polymer synthesis and functionalization with PEG. In Figure 2, the ^13^C-NMR spectra of pure PEUA and PEUA-PEG obtained with a 5:1 molar ratio are reported. 

As far as the ^13^C-NMR spectrum of PEUA is concerned, the signal at 153 ppm was attributed to the urethanic C=O, the signal at 136 ppm to MDI-C_1_ and MDI-C_4_, the signals at 120 ppm and 130 ppm to MDI-C_2_ and MDI-C_3_, respectively, and the signal at 41 ppm to the CH_2_ between the phenyl groups. The signals of the PPO *soft* phase were found at 18 ppm (methyl), 77 ppm (methine) and 73–75 ppm (methylene). The signals of DHMPA were at 46 ppm (quaternary carbon), 72 ppm (–CH_2_–), and ca. 20 ppm (–CH_3_). Finally, at 175 ppm, the signal of the carboxylic group was observed. As far as the ^13^C-NMR spectrum of PEUA-PEG is concerned, even if it was not possible to observe the peak related to the ester C=O, a new signal at 62 ppm was present, specifically the ester CH_2_ of the linked PEG. Additionally, the peak of the DHMPA quaternary carbon shifted from 46 ppm to 50 ppm, presumably due to the different chemical environments.

**Figure 3:**
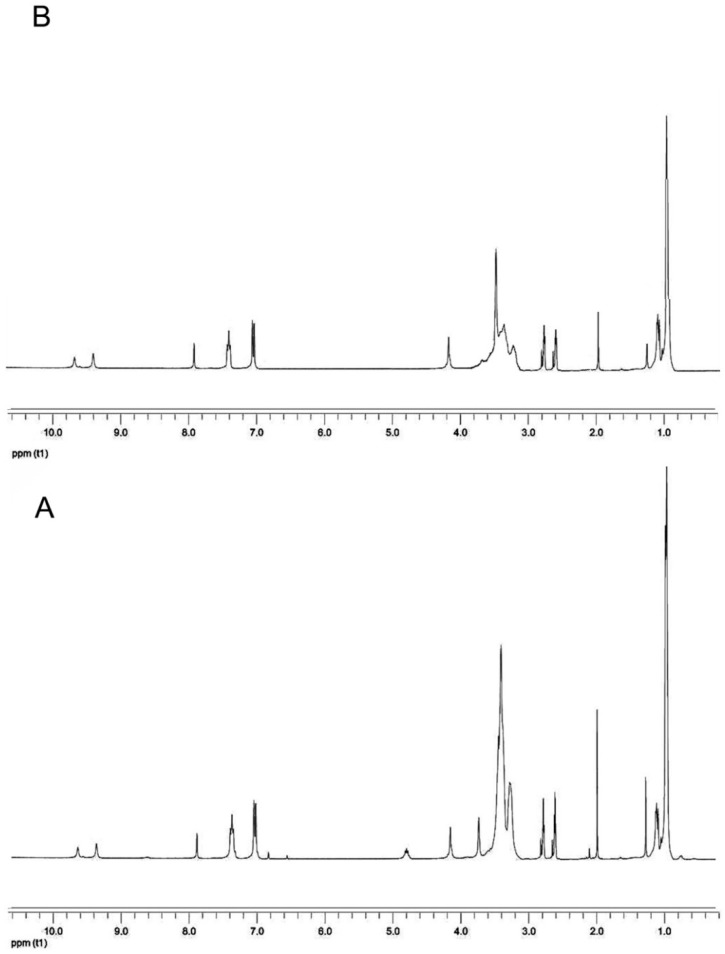
^1^H-NMR spectrum of PEUA (**A**) and PEUA-PEG obtained with a 5:1 PEG:PEUA molar ratio (**B**).

In Figure 3A, the ^1^H-NMR spectrum of PEUA is reported. The peaks at 9.6 and 10 ppm were attributed to urethane (NH), and the signal splitting was presumably due to the different linkers (PPO or DHMPA). The signals in the range of 7–8.0 ppm were attributed to the MDI aromatic ring, and the signal at 3.8 ppm was related to –CH_2_– between the two phenyls. The PPO methine and methylene groups showed a wide peak at 3.4 ppm when linked to the ether oxygen, and at 4.9 ppm when linked to the urethane NH. The signal at 3.5 ppm was attributed to the DHMPA methylene groups while the signal at 1.1 ppm was related to the methyl groups of DHMPA and PPO.

The signals of the PEG –CH_2_– groups in PEUA-PEG are in the same spectral range (ca. 3.6 ppm) as the protons of PPO (Figure 3B). The yield of functionalization was calculated by the ratio of the intensity of the peak at 7 ppm (aromatic ring), and that of the peak at 3.6 ppm (methylene groups), from which the contribution of PPO was subtracted. The resulting yield of esterification was ca. 30% in the case of the polymer obtained with a 5:1 PEG:PEUA molar ratio, and 20% for the soluble portion of the polymer obtained with a 3:1 molar ratio. 

**Figure 4:**
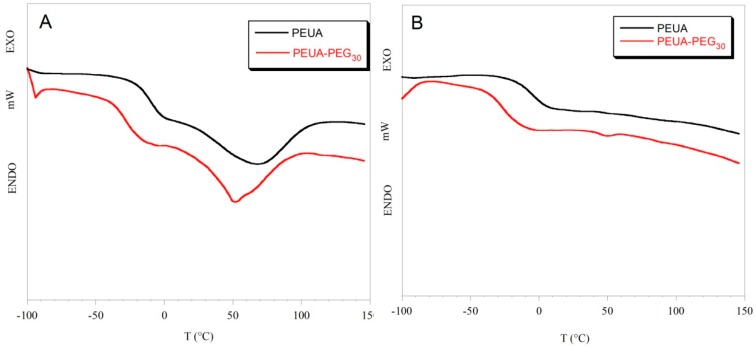
Thermograms of PEUA and PEUA-PEG_30_ in the I cycle (**A**) and the II cycle (**B**) of heating.

In segmented polyurethanes, differential scanning calorimetry can allow for not only the determination of the polymer transition temperatures, but also the estimation of polymer *hard/soft* phase segregation. Specifically, information on polymer phase segregation can be extrapolated by the glass transition temperature (T_g_), which, being characteristic of the *soft* phase of the polymer, depends on the *soft* phase mobility, and is affected by the interaction of the *soft* phase with the *hard* phase. An increase in the T_g_ value usually suggests low mobility of the *soft* phase, as a consequence of an increase in *hard/soft* phase mixing [46]. In Figure 4, as an example, the thermograms of PEUA and PEUA-PEG_30_ in cycles I and II of heating are reported, while in Table 1, the values of the glass transition temperature, and the variation of the specific heat (ΔC_p_) at T_g_ for all of the polymers are reported. The T_g_ value of free PPO, found in the literature [47], is also reported in Table 1 for comparison.

In the first cycle of heating, a flex, corresponding to the glass transition of the *soft* phase, and a broad endothermic band, corresponding to the melting of *hard* microdomains, can be observed in the thermograms of all polymers. Such an endothermic transition was no longer observable in the II cycle of heating. The T_g_ values of the PEG-functionalized polyurethanes were similar and lower than that of PEUA (Table 1). That suggests a greater phase segregation in the PEG-functionalized polyurethanes, compared to pristine PEUA. Presumably, PEG chains introduced in the *hard* phase of PEUA favored the cohesion of the *hard* domains by hindering the establishment of H-bonds among the urethane –NH groups of the *hard* phase and the ether oxygens of the *soft* phase. Additionally, some cross-linking between the polymer *hard* segments could occur, and further contribute to the segregation of the PEG-functionalized polymers. The variation in specific heat in correspondence with glass transition is also lower in the PEG-functionalized polymers compared to PEUA (Table 1), suggesting that a lower amount of the *soft* phase is involved in the glass transition. Presumably, a higher cohesion of the *hard* micro-domains, or the partial cross-linking of *hard* segments in the PEG-functionalized polymers can reduce the amount of the mobile portion of the *soft* phase. Such a phenomenon has already been described in the literature for segmented polyurethanes functionalized with carboxylate or sulfonate ions [48].

**Figure 5:**
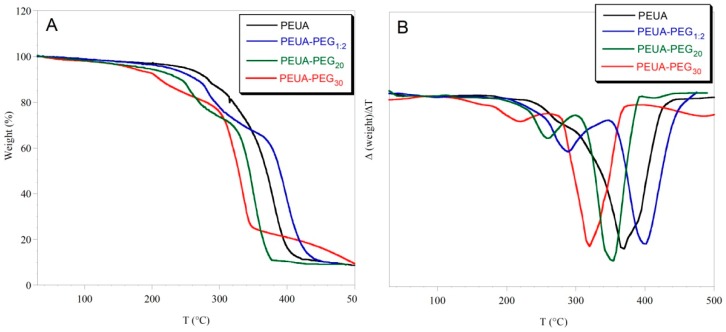
Thermogravimetric curves of PEUA, PEUA-PEG_1:2_, PEUA-PEG_20_, and PEUA-PEG_30_ (**A**); first derivative (Δ(weight)/ΔT) of the thermogravimetric curves (**B**). The subscript 1:2 on PEUA:PEG_1:2_ indicates the molar ratio employed during functionalization.

In Figure 5, the thermogravimetric curves of the pristine and functionalized polymers are reported. The first derivative of the weight vs. temperature is also reported. As it can be observed, PEUA-PEG_1:2_ (obtained with a PEG:PEUA molar ratio 2:1) was the most thermally stable polymer, presumably because of the higher crosslinking content. Pristine PEUA degraded mainly in one step from 250 to 400 °C, while the PEG-functionalized polymers showed two steps of degradation. The first one was attributed to PEG weight loss, and the second one to the degradation of the polyurethane backbone. By taking into consideration the weight of the PEUA repeat unit, a good agreement between the PEG weight loss and the 30% esterification yield determined by ^1^H-NMR was found for PEUA-PEG_30_. That was not true for PEUA-PEG_20_, presumably because of the chemical heterogeneity between the polymer soluble portion submitted to ^1^H-NMR, and the whole-polymer sample (soluble portion + insoluble portion) submitted to thermogravimetric analysis.

Material surface hydrophilicity/hydrophobicity is a very important feature that affects bacterial adhesion to the material itself. In general, it has been recently shown that superhydrophobic and superhydrophilic surfaces can both prevent microbial adhesion [49]. Superhydrophobic surfaces are those resembling the lotus leaf, which has been shown to have a water contact angle that is higher than 150°, and self-cleaning properties, thanks to its hierarchical micro/nanostructured surface covered with a low surface energy waxy hydrophobic film [50]. On the other side, very hydrophilic surfaces have intrinsic antifouling properties, due to the formation of a dense layer of water molecules, which weakens the interaction between the bacterial cell surface and the material surface [29,51]. 

To evaluate the effect of PEG-grafting on polymer hydrophilicity, polymer swelling in water and the dynamic contact angle were evaluated. Since these analyses, as well as the subsequent mechanical and biological tests, were performed on polymer films obtained by solvent casting (see Experimental Section), only the soluble polymers PEUA and PEUA-PEG_30_ will be subsequently characterized.

**Figure 6:**
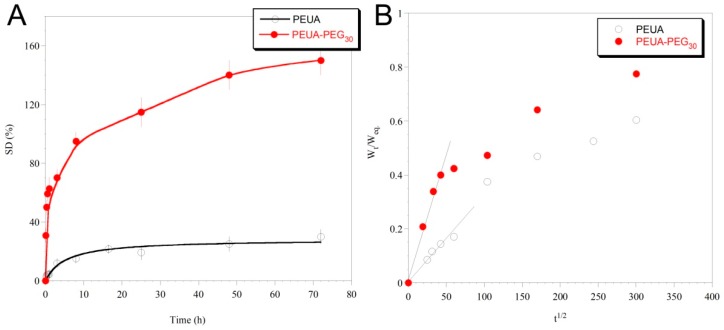
Swelling curves of PEUA and PEUA-PEG_30_ (**A**); Ratio between the swollen polymer mass at time *t*, and at the equilibrium (W_t_/W_eq_), as a function of the ratio of the square root of time (**B**). The diffusion coefficient was extrapolated by the slope of the linear fitting of the initial points.

In Figure 6A, the swelling curves for the two polymers are reported. 

As it can be observed, PEUA-PEG_30_ was more strongly hydrophilic than PEUA, reaching a swelling degree at an equilibrium of ca. 150%, compared to ca. 25% of PEUA. Such a big increase in polymer hydrophilicity after PEUA functionalization with PEG is surely due to the PEG intrinsic hydrophilic properties, but presumably also to the good *hard/soft* phase segregation of PEUA-PEG_30_, which makes the polymer *soft* phase free to move and interact with water molecules. This hypothesis was confirmed by the calculation of the Diffusion Coefficient (D) of water in each of the two polymeric matrices. Specifically, in a diffusional regime in which the Fick’s first law is valid, D can be calculated by the following equation [52]: $\frac{W_{\mathrm{tot}}}{W_{\mathrm{tot}}^{\infty }}=\frac{4t^{1/2}D^{1/2}}{L\pi^{1/2}}$ where *W_eq_* is the amount of water adsorbed at the equilibrium, *W_t_* is the amount of water that is adsorbed at the specific time *t*, and *L* is the polymer film thickness (which was assumed to be constant during swelling). This expression describing the transport behavior of the penetrant into the polymer can be applied only to the initial stages of swelling, i.e., up to a 60 % increase in the mass of the polymer (W_t_/W_eq._ ≤ 0.6) [53]. The values of D were obtained from the plot of the ratio of the swollen polymer mass at time *t*, and *t* = eq. (W_t_/W_eq_), as a function of the ratio of the square root of time (*t*^1/2^), by following the initial slope method (Figure 6B). The following D values were extrapolated: D_PEUA_ = (1.7 ± 0.1) × 10^−9^ cm^2^∙ s^−1^, and D_PEUA-PEG30_ = (5.0 ± 0.2) × 10^−9^ cm^2^ ∙s^−1^. The higher D value of water in PEUA-PEG_30_ compared to PEUA suggests a higher ability of water molecules to diffuse into the polymer matrix, presumably due to a higher mobility of the hydrophilic *soft* phase achieved by the higher polymer *hard/soft* phase segregation. 

**Figure 7:**
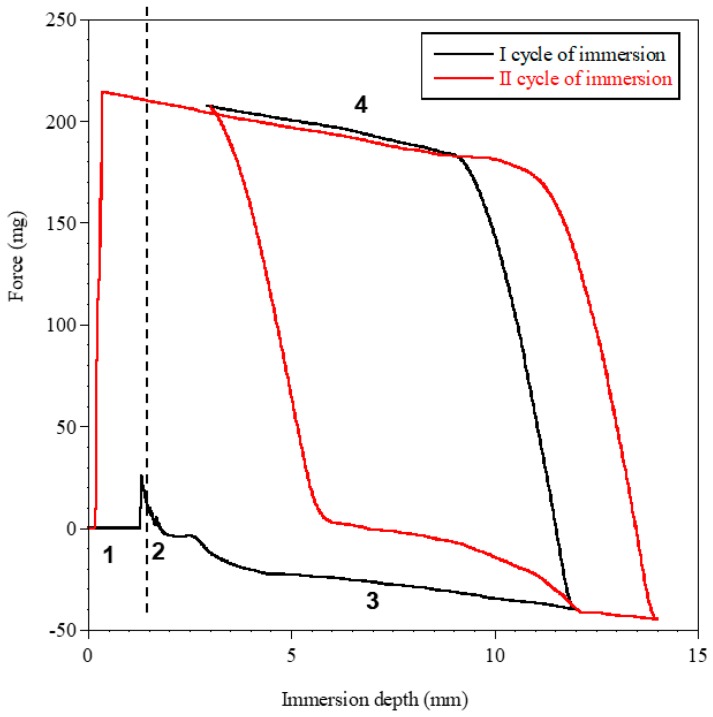

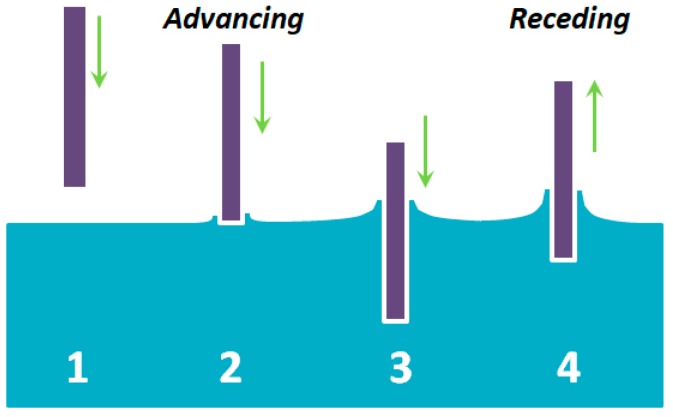
DCA cycles of immersion for PEUA-PEG_30_ by using the Wilhelmy plate method. In the figure, numbers 1 to 4 indicate the position of the plate with respect to the liquid, as shown in the image below the figure. 1—Out of the liquid; 2—point of touch of the sample with the liquid; 3—Immersion into the liquid (θ_adv_); 4—Emersion from the liquid (θ_rec_).

Measurements of dynamic contact angle confirmed the higher hydrophilicity of PEUA-PEG_30_, compared to PEUA. In such an analysis, the surface hydrophilicity (or wettability) can be evaluated [54]. In Figure 7, as an example, the profile of the two immersion cycles for PEUA-PEG_30_ is shown, and in Table 2, the values of the contact angles, in advancing and in receding, and the contact angle hysteresis, are reported. As observed in Table 2, in the first cycle of immersion, the two polymers showed similar θ_av_, suggesting a similar wettability. However, PEUA-PEG_30_ showed a significantly lower θ_rec_ than PEUA (35 ° vs. 47 °) and, as a consequence, a higher hysteresis. 

Such a finding is presumably related to the surface chemical heterogeneity of PEUA-PEG_30_ (greater than PEUA). Additionally, in the second immersion cycle, PEUA-PEG_30_ showed a kinetic contact angle hysteresis, evidenced by the decrease of θ_adv_ from 94 ° to 83 °, suggesting a molecular rearrangement of the polymer surface on wetting, which presumably involved the exposition of the polar PEG chains towards water. 

Langmuir firstly observed the flipping of the surfactant molecules, as polar head groups migrated towards hydrophilic environments [55]. It was, later demonstrated that such re-organization was driven by the minimization of the surface free energy at the interface [56].

**Figure 8:**
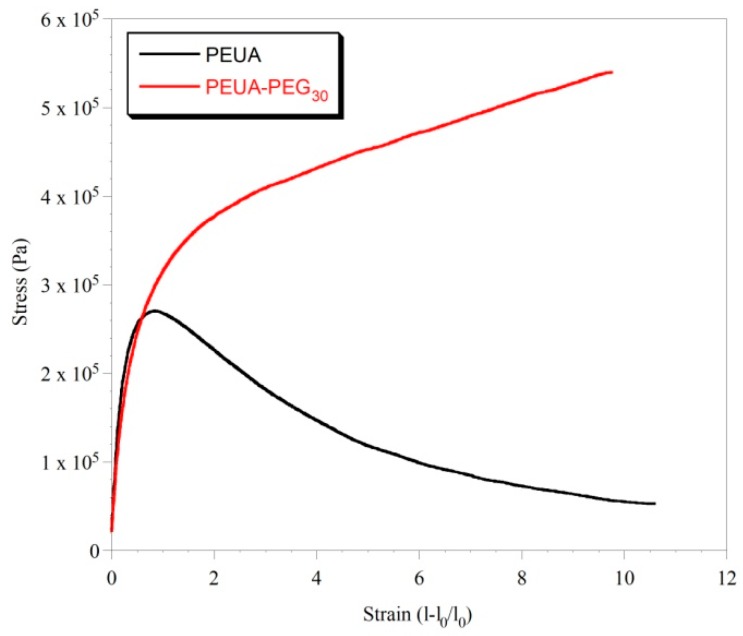
Stress-strain curves of PEUA and PEUA-PEG_30_.

The two polymers also showed significantly different mechanical properties, as shown by INSTRON analysis. In Figure 8, the stress–strain curves of PEUA and PEUA-PEG_30_ are reported.

As it can be observed, both polymers showed an elongation at a break of ca. 10. The Young Modulus, determined from the slope of the initial linear trend, was also similar for the two polymers (E_PEUA_ = 0.9 MPa e E_PEUA-PEG30_ = 0.7 MPa). However, the trend of the two stress–strain curves was very different. Indeed, PEUA showed a Yield Point at an elongation of ca. 0.7, in correspondence with which a collapse of the material resistance was observed. In contrast, PEUA-PEG_30_ showed a typical trend of elastomeric materials, in which the Yield Point was not really clear, and the stress increased with the elongation up to material break. The stress at break of PEUA-PEG_30_ (0.5 MPa) is also ca. one order of magnitude higher than that of PEUA (0.06 MPa). Overall, the mechanical properties of PEUA-PEG_30_ are coherent, with a marked *hard/soft* phase segregation in this polymer as evidenced by DSC analysis. Presumably, the presence of strong chain–chain interactions hinders chain slippage, and make the material resistant to big deformations. In addition, the occurrence of chemical cross-linkages between the *hard* segments cannot be excluded, even if the solubility of the sample suggests a case of low cross-linking. Of course, if present, *hard–hard* segment cross-linkages can further justify the elastomeric properties of the polymer. 

**Figure 9:**
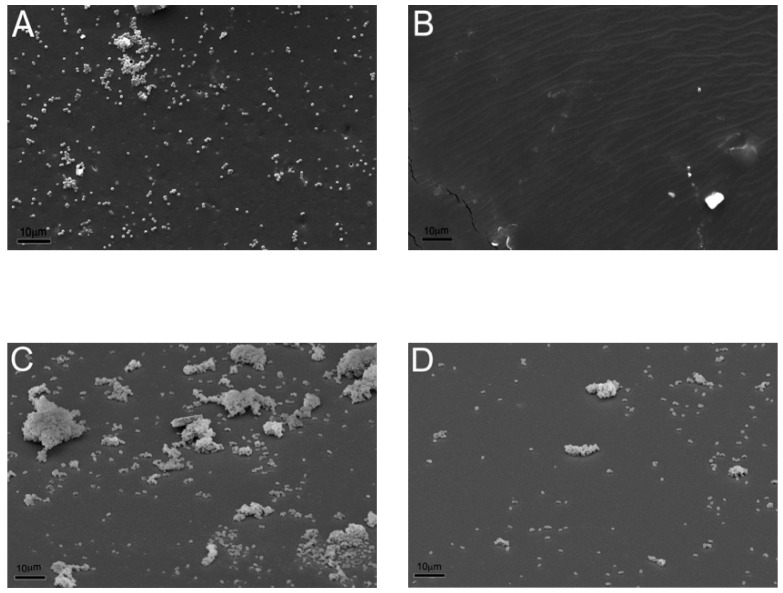
Initial bacterial adhesion after 30 min incubation on PEUA (**A**) and PEUA-PEG_30_ (**B**). Biofilm formation after 24 h incubation on PEUA (**C**) and PEUA-PEG_30_ (**D**). Scale bar = 10 µm.

Finally, the abilities of PEUA and PEUA-PEG_30_ to prevent microbial adhesion and biofilm formation was evaluated. In Figure 9, SEM micrographs showing the surface of PEUA and PEUA-PEG_30_ after 30 min (Figure 9A,B) or 24 h (Figure 9C,D) incubation with *S. epidermidis* suspensions are reported. 

As it can be noted, *S. epidermidis* can easily adhere to PEUA. Indeed, the presence of *S. epidermidis* colonies in the first stages of adhesion could be observed on the PEUA surface (Figure 9A). In contrast, PEUA-PEG_30_ was essentially free from colonization (Figure 9B). Such different initial bacterial adhesion rates were reflected in the biofilm formations on the two polymer surfaces. Indeed, heavy colonization of the PEUA surface was observed with the presence of large biofilm structures (Figure 9C) after 24 h incubation, while just a few sporadic adhering cells and small bacterial aggregates were present on PEUA-PEG_30_ (Figure 9D). Such difference in biofilm formation was confirmed by the cell count, the number of CFU per surface unit (CFU/cm^2^) being 4 × 10^8^ and 7 × 10^5^ for PEUA and PEUA-PEG_30_, respectively. 

The excellent ability of PEUA-PEG_30_ to reduce bacterial adhesion and biofilm formation is definitely related to its higher bulk and surface hydrophilicity which also presumably involve the exposition of PEG chains at the material/water interface, as shown by dynamic contact angle analysis. However, since the polymer does not completely inhibit bacterial adhesion, such a system should be used in combination with other anti-biofilm molecules, to maximize its performance [15,57,58]. Unexpectedly, the mechanical resistance of the PEG-functionalized polymer also improved because the presence of PEG, with benefits for the application. 

## 3. Materials and Methods

### 3.1. Materials

Polyethylene glycol (PEG, M_n_ = 1000 g/mol) (Sigma Aldrich s.r.l., Milan, Italy) was used as received. Methylene bis-phenyl-diisocyanate (MDI) (Sigma Aldrich s.r.l.) was distilled before use. Polypropylene oxide (PPO) (1200 g·mol^−1^, Sigma Aldrich s.r.l.) was degassed under vacuum at 60 °C for 12 hr. Di-hydroxymethyl propionic acid (DHMPA) (Sigma Aldrich s.r.l.), dicyclohexylcarbodiimide (DCC) (Sigma Aldrich s.r.l.) and 4-dimethylaminopyridine (DMAP) (Sigma Aldrich s.r.l.) were used as received.

### 3.2. Polymer Synthesis and Functionalization

A carboxylated segmented polyurethane, PEUA (one carboxyl group per repetitive unit, Mw = 40,000 g·mol^−1^, M_w_/M_n_ = 1.6, Figure 1) was synthesized by a two-step condensation of MDI, PPO, and DHMPA in a 2:1:1 stoichiometric ratio, as previously described [43].

Functionalization of PEUA with PEG was achieved by a DCC-activated esterification of polymer carboxylic acids, by using DMAP as an accelerator [59]. Specifically, DMAP and DCC, in equimolecular amounts with respect to the polymer carboxyl groups, were added to a 5% (*w*/*v*) solution of PEUA in dichloromethane at 0 °C. After 20 min, PEG was added in molar excess (2:1, 3:1 and 5:1) with respect to PEUA to avoid polymer crosslinking. Temperature was then raised to 25 °C and the solution was left under stirring for 24 hr. After the reaction, dicyclohexylurea precipitate during polymer functionalization was filtered. Then, the polymer was recovered by precipitation in hexane, and washed in water to eliminate the unreacted PEG. The polymer, whose repetitive unit is reported in Figure 1, was named PEUA-PEG_X,_ where x is the functionalization degree.

### 3.3. Polymer Characterization

^1^H-NMR and ^13^C-NMR spectra were performed, employing a Varian XL 300 instrument (Varian Inc., Palo Alto, CA, USA) and deuterated di-methylformamide (DMF-d7) as the solvent.

Differential scanning calorimetry (DSC) was performed from −100 to +150 °C under N_2_ flux by using a Mettler TA-3000 DSC apparatus (Mettler Toledo, Columbus, Ohio, US). The scan rate used for the experiments was 10 °C·min^−1^ and the sample weight was 6–7 mg. Thermo-gravimetric analysis (TGA) was carried out employing a Mettler TG 50 thermobalance at a heating rate of 10 °C·min^−1^ under N_2_ flow in the temperature range 25–600 °C.

Swelling measurements of both the pristine and functionalized polyurethanes were performed at room temperature in water. For such experiments, circular test samples (1 cm in diameter and 100 µm in thickness) were obtained by cutting cast films of the polymers. Polymer films were obtained by dissolving the polymer in tetrahydrofuran (5% *w*/*v*) and layering such a solution on circular Teflon plates, 5 cm in diameter. After solvent evaporation at room temperature, polymer films were detached from the Teflon plates with the aid of tweezers. The polyurethane samples were then immersed in water. At the determined times, specimens were removed from the water and weighed, after removal of the excess of the solvent using filter paper. The analysis was repeated until a constant weight (equilibrium swelling) was reached. The swelling degree, SD, was calculated by applying the following equation:

$$SD\;\left(\%\right)=\frac{W_{t}-W_{0}}{W_{0}}$$

where *W_t_* is the weight of the sample after swelling at time *t*, and *W_0_* was the initial weight of the film. Five parallel swelling experiments were performed for each sample, and data were reported as the average value ± standard deviation.

The dynamic contact angle was performed by using a Dynamic Contact Angle Analyzer Cahn DCA 312 (CAHN Instruments, Inc, South Carmenita Road, Cerritos, CA, USA), on the basis of the Wilhelmy balance method. Such a method consists of detecting by a balance the change in the weight of a thin vertical plate when it is brought in contact with a liquid. The detected weight change is a combination of the buoyancy and the wetting force, while the gravity force remains the same. Since the wetting force (*F_w_*) is defined as:

F_w_ = γ_lv_ p cosθ

where *γ_lv_* is the liquid surface tension, *p* is the perimeter of the sample, and *θ* is the contact angle, the force change (*F*) detected by the balance is:

F = γ_lv_ p cosθ − VΔρ g

where *V* is the volume of the displaced liquid, *Δρ* is the difference in density between the liquid and air, and *g* is the acceleration of gravity. Thus, as long as *γ_lv_* and the solid perimeter (*p*) are known, the contact angle value can be determined. 

Contact angle measurements were performed with an immersion rate of 20 µm/s, carrying out two consecutive immersion cycles. In the second immersion cycle, the immerged area of the sample was greater than that of the first cycle, in order to verify the absence of the release of substances from the sample into water. The analyses were performed on samples obtained by layering the polymer on rectangular glass coverslide (1 cm × 2 cm) by solvent casting. When the sample was immersed into the liquid, the advancing contact angle (θ_adv_) was recorded, while when the sample was emerging, the receding contact angle (θ_rec_) was measured (Figure 8). The difference between the advancing and the receding angles is called the contact angle hysteresis (H = θ_adv_ − θ_rec_), which mainly arises from surface roughness and/or chemical heterogeneity [60]. However, other factors, including liquid adsorption and retention, or molecular rearrangement on wetting, can contribute to hysteresis [61]. 

The mechanical properties of the pristine and functionalized polyurethanes were studied by tensile tests, which were carried out with an ISTRON 4502 instrument (Instorn Inc., Norwood, MA, USA). Measurements were performed on rectangular polymer films (50 mm long × 6 wide × 0.2 thick), by employing a 10 N load cell and a deformation rate of 50 mm·min^−1^.

### 3.4. Bacterial Strains and Culture Medium

For an evaluation of the antifouling features of polymers, the oxacillin-resistant *Staphylococcus epidermidis* ATCC 35984, a strong exopolysaccharide producer [62], was routinely grown in tryptic soy agar (TSA) and tryptic soy broth (TSB). Biofilm production was assessed according to the protocol described by Francolini et al. [63]. 

### 3.5. Assessment of the Polymer Ability to Prevent Bacterial Adhesion and Biofilm Formation

To evaluate the effect of polyurethanes on bacterial adhesion and biofilm formation, test tubes were filled with 2.5 mL of a bacterial suspension (0.5 McFarland) and grown at 37 °C in TSB supplemented with 1% glucose, to promote massive exopolysaccharide production. One tube was used as a reference (control), while polymer discs (1 cm in diameter, 100 μm in thickness) were introduced into the other tubes. Tubes were incubated at 37 °C for 30 min to assess early bacterial adhesion, or for 24 h to assess late bacterial adhesion [64,65].

After incubation, bacterial adhesion and biofilm formation were assessed by scanning electron microscopy (SEM, Assing, Rome, Italy). Specifically, polymer discs were collected, washed twice with PBS (pH 7.4) to remove loosely adherent cells, fixed with 2.5% glutaraldehyde in 0.1 M cacodylate buffer (pH 7.4) at room temperature for 30 min, dehydrated through graded ethanol, treated with hexamethyldisilazane for 20 min, and gold-sputtered for SEM observation.

Polymer discs incubated with bacterial suspension for 24 h (biofilm formation) were also submitted to colony forming unit (CFU) counts. Specifically, disks were put into test tubes with 10 mL of phosphate buffer, and sonicated for 5 min to remove the adherent cells. Six 10-fold dilutions were prepared, and three 10 μL aliquots of each dilution were plated onto TSA plates. Plates were then incubated at 37 °C for 18 hr, and CFUs were counted and referred to the polymer surface unit (CFUs/cm^2^).

### 3.6. Statistics

Analysis of variance comparisons were performed using Mini-Tab. Differences were considered significant for *p* < 0.05. Data are reported as means ± SD. 

## 4. Conclusions

This study confirms the strong antifouling ability of polyethylene glycol materials, and shows the great potential for PEG-grafting to confer bacterial resistance properties to segmented polyurethanes. Indeed, the functionalization of a thermoplastic polyurethane with PEG resulted in a material with superior elastomeric properties, and the ability to prevent the adhesion of the Gram-positive *S. epidermidis*, a microbial pathogen that is commonly isolated in medical device-related infections. Additionally, since the developed antifouling material is intrinsically active, it does not exhaust its activity over time, and it could provide long-term protection, at least in principle. Under an applicative point of view, the PEG-functionalized polyurethane obtained in this study could find a role in several biomedical applications, spanning from intravascular medical device manufacturing to wound dressings, where it could be applied as an antifouling coating, or incorporated within the bulk structure.

## Figures and Tables

**Table 1:** Glass transition temperature (T_g_) and variation of specific heat (ΔC_p_) at the glass transition temperature for PEUA and PEG-functionalized polyurethanes. The glass transition temperature of the free *soft* phase (PPO) is also reported, for comparison. ^(*)^ The subscript indicates that the PEUA:PEG molar ratio employed during functionalization. Indeed, it was not possible to determine the functionalization degree for this polymer, as it is insoluble in common solvents.

Polymer	T_g_ (°C)	ΔC_p_ (J/g*K)
PEUA	−11 ± 2	0.50 ± 0.02
PEUA-PEG_1:2_^(*)^	−33 ± 2	0.39 ± 0.02
PEUA-PEG_20_	−29 ± 2	0.40 ± 0.03
PEUA-PEG_30_	−27 ± 2	0.43 ± 0.03
*Soft* phase (PPO)	−67	-

**Table 2:** Contact angle values (θ_adv_ and θ_rec_) and hysteresis (H) for PEUA and PEUA-PEG_30_ in the first and second cycles of immersion.

Polymer	I cycle		II cycle		H (I cycle)	H (II cycle)
	Θ_adv_	Θ_rec_	Θ_adv_	Θ_rec_		
PEUA	93 ± 3	47 ± 3	92 ± 1	50 ± 3	46	42
PEUA-PEG_30_	94 ± 2	35 ± 3	83 ± 2	37 ± 1	59	46
